# Efficacies of Sodium Hypochlorite and Quaternary Ammonium Sanitizers for Reduction of Norovirus and Selected Bacteria during Ware-Washing Operations

**DOI:** 10.1371/journal.pone.0050273

**Published:** 2012-12-05

**Authors:** Lizanel Feliciano, Jianrong Li, Jaesung Lee, Melvin A. Pascall

**Affiliations:** Department of Food Science and Technology, The Ohio State University, Columbus, Ohio, United States of America; Columbia University, United States of America

## Abstract

Cross-contamination of ready-to-eat (RTE) foods with pathogens on contaminated tableware and food preparation utensils is an important factor associated with foodborne illnesses. To prevent this, restaurants and food service establishments are required to achieve a minimum microbial reduction of 5 logs from these surfaces. This study evaluated the sanitization efficacies of ware-washing protocols (manual and mechanical) used in restaurants to clean tableware items. Ceramic plates, drinking glasses and stainless steel forks were used as the food contact surfaces. These were contaminated with cream cheese and reduced-fat milk inoculated with murine norovirus (MNV-1), *Escherichia coli* K-12 and *Listeria innocua.* The sanitizing solutions tested were sodium hypochlorite (chlorine), quaternary ammonium (QAC) and tap water (control). During the study, the survivability and response to the experimental conditions of the bacterial species was compared with that of MNV-1. The results showed that current ware-washing protocols used to remove bacteria from tableware items were not sufficient to achieve a 5 log reduction in MNV-1 titer. After washing, a maximum of 3 log reduction in the virus were obtained. It was concluded that MNV-1 appeared to be more resistant to both the washing process and the sanitizers when compared with *E. coli* K-12 and *L. innocua.*

## Introduction

Norovirus is the leading cause of epidemic gastroenteritis and the major cause of foodborne illness in the United States. It is responsible for at least 50% of all gastroenteritis outbreaks worldwide [1]. Norovirus is highly contagious and only a few particles are sufficient to cause illness [2], [3]. Transmission of the vast majority of foodborne norovirus infections is considered to be through the oral–fecal route, either by direct person-to-person spread or indirectly through contaminated food or water [4], [5]. Common foods associated with the transmission of norovirus include fresh produce, ready-to-eat foods, oysters, baked goods, and berries [6]. Ingestion of aerosolized vomitus, indirect exposure via fomites or contaminated environmental surfaces are also recognized as important means for the transmission of noroviruses [1].

Restaurants and foodservice establishments are recognized as important sites for the transmission of foodborne illnesses [7], [8]. As part of a 10 year study which began in 1998, the Food and Drug Administration (FDA) collected data from more than 800 food establishments. This study evaluated the occurrence of practices and behaviors commonly identified by the Centers for Disease Control and Prevention (CDC) as contributing factors to foodborne illness outbreaks [9]. At the conclusion of the study, five foodborne illness risk factors were identified. These risk factors include: food from unsafe sources; poor personal hygiene; inadequate cooking; improper holding temperatures; and contaminated equipment. The report also showed that contaminated equipment had a high percentage of out of compliance observations. Under this category, improper cleaning and sanitizing of food-contact surfaces was the item most commonly observed to be out of compliance.

A previous study conducted by Handojo et al., (2009) [10], showed that traditional sanitizers were able to reduce *Escherichia coli* K-12 and *Staphylococcus epidermidis* by ≥5 log, the minimum reduction required by the FDA Food Code for an effective sanitization protocol for bacteria [11]. As a result, these organisms were used as comparisons with that of murine norovirus under similar conditions. These bacterial species were used as surrogates for *E. coli* O157:H7 and *L. monocytogenes* (pathogens of current public health concern). However, little information regarding the efficacy of traditional sanitizers for the reduction of foodborne viruses from food contact surfaces is available in the literature. Thus, the objective of this study was to evaluate the sanitization efficacy of quaternary ammonium and sodium hypochlorite for the reduction of murine norovirus (a human norovirus surrogate) on different contaminated tableware items using normal ware-washing protocols (manual and mechanical). At the end of this study this objective was met.

## Materials and Methods

### Cell culture and virus stock

Murine norovirus (MNV-1) was provided by Dr. Herbert Virgin IV from Washington University School of Medicine. The preparation and infectious titer assays for MNV-1 were performed using the murine macrophage cell line RAW 264.7 (ATCC, Manassas, VA), as described by Wobus et al., (2006) [12], with minor modifications. The cells were cultured and maintained in 150 cm^2^ tissue culture flasks (BD Falcon, Bedford, MA) containing high-glucose Dulbecco's Modified Eagle Medium (DMEM; Invitrogen, Carlsbad, CA) supplemented with 10% fetal bovine serum (FBS; GIBCO-Invitrogen, Grand Island, NY) at 37°C and 5% CO_2_ atmosphere. Confluent RAW 264.7 cells were infected with the MNV-1 at a multiplicity of infection (MOI) of 0.2. The flasks were incubated at 37°C and 5% CO_2_ for 1 h, with agitation every 15 min. After 1 h incubation, DMEM supplemented with 2% FBS was added to the flask and incubated at 37°C and 5% CO_2_ for 48 h. When extensive cytopathic effect (CPE) was observed, the virus was harvested by freeze-thawing three times at these temperatures −80°C and 37°C, respectively, to lyse the cells and release virus particles. The purification of MNV-1 was performed by transferring the cell-virus suspensions into 50 ml sterile conical centrifuge tubes (USA Scientific, Ocala, FL) and centrifugation at 3,000 rpm for 20 min using an Allegra 6R centrifuge with a GH-3.8 swinging bucket rotor (Beckman Coulter, Brea, CA). The supernatant was collected and stored at 4°C for immediate use or at −80°C in aliquots for future use. The initial titer of MNV-1 stock was 10^8^ plaque forming unit (PFU)/ml.

### Preparation of bacterial cultures

*E. coli* K12 (ATCC 29181) and *L. innocua* (ATCC 33090) were stored in a −80°C freezer in 30% (v/v) sterile glycerol (Fisher Scientific, Fair Lawn, NJ) and revived when required for the experimental procedure. The stock culture of each organism was prepared by transferring a loopful of the *E. coli* and *L. innocua* into 50 ml of Trypticase soy broth (Difco, Becton Dickinson, Sparks, MD) containing 0.3% (wt/wt) yeast extract (Fisher Scientific) (TSBYE) and then incubation of the cultures at 37°C for 24 h. A loopful of this broth was then inoculated into a Trypticase soy agar slant (Difco, Becton Dickinson) supplemented with 0.3% (wt/wt) yeast extract (TSAYE) and incubated at 37°C for 24 h. This TSAYE containing the cell cultures was stored in a refrigerator at 4°C and used as a stock culture.

Prior to each experiment, a loopful of either *E. coli* K12 or *L. innocua* stock culture was propagated aerobically in 100 ml TSBYE at 37°C for 24 h. Each cell broth was centrifuged (Kendro Laboratory Products, Sorvall RC 5C Plus, Newtown, CT) at 6,000 rpm for 10 min at 4°C. The supernatant was decanted and the cell suspension was resuspended in 100 ml of 0.1 M potassium phosphate buffer (pH 7.2) until an initial concentration of approximately 1.0×10^9^ CFU/ml for both *E. coli* K12 and *L. innocua* was achieved. Each cell suspension was separately mixed with the food samples to be tested.

### Food samples preparation and inoculation

Dairy products are known for being difficult to remove from various surfaces, especially when dried [13]. Therefore, cream cheese spread and 2% reduced fat ultra high temperature (UHT) milk were used to contaminate the food contact surfaces in this study to simulate a worst case scenario. These were purchased from a local grocery store in Columbus, OH. They were both stored in a refrigerator at 4°C until ready to use. Ceramic plates, stainless steel forks and drinking glasses were the tableware items tested. These were sterilized by autoclaving at 121°C for 20 min before each experiment.

### Ceramic plates

For the contamination of the ceramic plates, 90 g of cream cheese were weighted in a sterile beaker, heated for less than 10 sec in a microwave, inoculated with 10 ml of the virus stock (1∶10 w/w) to provide a final titer of approximately 7 log plaques forming units per gram (PFU/g). This was then stirred with a sterile tongue depressor (Fisher Scientific, Florence, KY) to ensure proper mixing of the virus with the cream cheese. A total of 3 g of this cream cheese was then applied to the entire food contact surface of each ceramic plate. The plates were air dried for 1 h at room temperature (25°C) on a flat, sterile rack prior to the washing protocol. The same procedure was followed for *E. coli* K- 12 and *L. innocua*.

### Forks and drinking glasses

From the contaminated cream cheese above, a 0.5 g aliquot was applied to the fork. For contamination of the glasses, 45 ml of milk were transferred to a 50 ml sterile conical tube and inoculated with 5 ml of virus stock solution (1∶10 v/v). From this solution, 0.5 ml was applied to the inner wall of each drinking glass. Both the forks and the glasses were then air dried for 1 h at room temperature (25°C) on a flat, sterile rack prior to the washing protocol. The same procedure was followed for *E. coli* K- 12 and *L. innocua*.

### Preparation of the detergents and sanitizing solutions

Ecotemp Ultra Klene detergent (Ecolab, Inc., St. Paul, MN) and Monsoon detergent (Ecolab, Inc., St. Paul, MN) were used for the mechanical and manual washing, respectively. Ultra Klene detergent was used at 3,000 ppm concentration. The concentration used for the Monsoon detergent was 100 ppm. These concentrations were recommended by the manufacturer, as per the Food Code requirements.

Sodium hypochlorite (chlorine-bleach) and quaternary ammonium compounds (QAC) were used as the sanitizing solutions. Tap water was used as a control sanitizer. The chlorine bleach, containing 6% sodium hypochlorite, was purchased from a local grocery store. The sodium hypochlorite solution used in this study was 200±20 ppm and this concentration was determined using a HI 95771 Chlorine Ultra High Range Meter (Hanna Instruments, Ann Arbor, MI). The QAC was an OASIS 146 Multi-Quat sanitizer manufactured by Ecolab, Inc. (St. Paul, MN). It was used at a concentration of 200 ppm as determined by a HYDRION QT-10 Quat test paper (QA Supplies, Norfolk, VA). The water hardness for both mechanical and manual washing procedures was determined to be less than 120 ppm and it was determined using a Water Quality Test Strip kit (Hach Co., Loveland, CO).

### Mechanical ware-washing and sanitization of contaminated tableware items

A Hobart LXiC Dishwasher was connected to a hot water line and had an incoming water pressure of 138 kPa. Prior to each experiment, the machine was cleaned with hot water (49°C) and filled with fresh water. To ensure the proper volume and concentration of the detergent during the washing cycles, the detergent (Ecotemp Ultra Klene detergent) was directly added to the water tank. The resulting water-detergent solution had a concentration of 3,000 ppm (v/v). During the washing cycle, the tableware items were automatically sprayed with the wash water for 76.5 s at a pressure and temperature of 138 kPa and 49°C, respectively. After the washing cycle, the tableware items were automatically sprayed with the QAC sanitizing solutions for 10 s at 49°C. Once the sanitizing cycle was completed, all tableware items were air dried for 1 h at 24±2°C. This entire cycle was repeated but with the chlorine sanitizer instead. The chlorine concentration was measured after spraying.

### Manual ware-washing and sanitization of contaminated tableware items

A three compartment sink manufactured by Eagle Group, Inc. (Clayton, DE), was used for the washing, rinsing and sanitizing of the tableware items. Prior to each experimental run, this dishwasher was thoroughly cleaned with hot water and refilled with fresh water and detergent/sanitizer. The tableware items were washed with 100 ppm of the Monsoon detergent at 43°C for 30 s, soaked in tap water during the rinsing at 24°C for 10 s, and sanitized by soaking them in one of the sanitizing treatments at 24°C for 30 s. The test was repeated for each sanitizer and tableware item. Rubber gloves were worn throughout the experiment.

**Figure 1:**
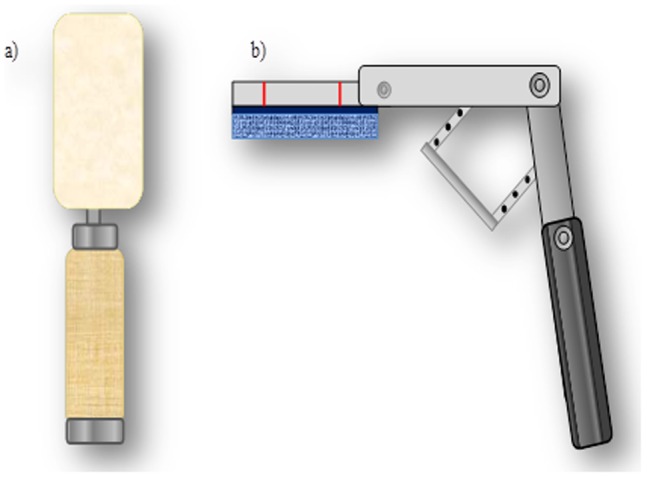
Devices used during manual ware-washing to clean the different tableware items. a) Cylindrical sponge b) Sponge attached to the spring-loaded tool.

During the washing step, each fork and ceramic plate was manually washed using a Scotch-Brite multi-purpose scrub sponge (3M, St. Paul, MN). A cylindrical device covered with a soft sponge was used to wash the drinking glasses (Fig. 1a). To ensure consistency of the force applied to remove the cream cheese from the plates and the forks during washing, the sponge was attached to a spring-loaded device (Fig. 1b). The forks were washed by using fifteen forward and fifteen backward strokes with the sponge. The plates and glasses were washed by using fifteen clockwise and fifteen counter-clockwise strokes. After washing, the tableware items were rinsed, sanitized and placed in a clean rack and air dried for 1 h at 24±2°C. The three compartments of the sink were washed with hot water (49°C) and bleach (10%) after each experimental run.

### Viral sampling of the tableware surfaces

The initial viral titer of the virus stock, as well as from the contaminated milk, was determined by transferring 200 µl from each to a test tube containing 1.8 ml of phosphate buffered saline (PBS). The titer of the contaminated cream cheese was determined by transferring a small amount of the cheese to a test tube containing 2 ml of PBS using a sterile calcium-alginate cotton-tipped swab (Fisher Scientific, Pittsburgh, PA). Prior to the ware-washing, 4 samples of each tableware item were collected after the air drying period. Once the sanitization step was completed and the tableware items air dried for 1 h, 4 samples of each item were collected. These samples (before and after the ware-washing procedure) were collected using cotton-tipped swabs moistened with the PBS solution. The test tubes containing the samples were vortexed to remove any viral particles attached to the tip of the swab. Serial dilutions (10-fold) of the samples were performed in PBS solution.

### Bacterial enumeration of the contaminated tableware surfaces and MNV-1 plaque assay

The quantification of the MNV-1 was performed by plaque assay. Confluent monolayers of RAW 264.7 cells were grown in 6-well plates (BD Falcon, Franklin Lakes, NJ) containing DMEM with 10% FBS for 24 h at 37°C and 5% CO_2_. After incubation, the growth medium was removed and the cell monolayers were infected with 500 µl of each sample dilution. The infected plates were incubated for 1 h at 37°C and 5% CO_2_, with agitation every 15 min. The plates were overlaid with 2 ml minimal essential medium (MEM) supplemented with 5% FBS, 1.6% sodium bicarbonate (7.5% [wt/v]), 0.5% penicillin-streptomycin (10,000 U of penicillin and 10,000 µg/ml streptomycin in 0.85% saline; GIBCO-Invitrogen), 2.5% HEPES, 1% glutamine, and 1.5% low-melting-point agarose (GIBCO-Invitrogen). After adding the overlay, the plates were placed in a refrigerator (4°C) for 1 h and then incubated at 37°C and 5% CO_2_ for 2 d. Following this, 2 ml of 10% formaldehyde in PBS solution were added to each well to fix the cells. The fixation was done for 4 h. The overlay-formaldehyde solution was removed and the wells stained with 0.05% crystal violet (wt/v) for 1 h in order to visualize and count the viral plaques.

For enumeration of the bacteria, the samples before and after the ware-washing procedure were collected using the cotton-tipped swabs, previously moistened in 0.1% peptone water solution. The swabs were then transferred to test tubes containing 0.1% peptone water and vortexed vigorously to remove any bacteria from the tips.

The total viable counts were determined by serial dilution of the samples using test tubes and then plated into TSAYE. The plates were incubated at 37°C for 36 h. A Darkfield Colony Counter (American Optical, Buffalo, NY) was used to count the bacterial cells. The detection limit for estimating the bacterial numbers was 2 CFU per tableware item.

### Inactivation of MNV-1 in suspension solutions

To determine the effectiveness of the sanitizers for inactivation of MNV-1 in solutions, MNV-1 stock (10^8^ PFU/ml) was directly inoculated into DMEM or milk, followed by addition of different sanitizers including chlorine and QAC at final concentration of 200 ppm for each sanitizer, respectively. After incubation at room temperature for 1 min, 50 µl of aliquots were subject to 10 time's serial dilutions. The survival of MNV-1 in both suspensions was quantified by plaque assay as described above.

### Statistical analysis

All tests were duplicated in this study. The viral titers were expressed as log PFU per tableware (surface). For the bacterial cells, the counts were expressed as log CFU per tableware. During each test, 4 tableware items were selected for viral/bacterial enumeration before and after sanitization. The reported values of the viral counts were the mean values of two trials ± standard deviations. Multifactor analysis of variance (ANOVA) was used to determine the significance between the mean values. The data analyses were performed by the General Linear Model function and Tukey's multiple comparison test with the SAS, version 9.2, statistical program (SAS Institute, Cary, NC) to determine the level of significance between the effect of each sanitizer, tableware item and ware-washing protocol (manual vs. mechanical). To properly identify any significant differences at low levels, the *p* value was set at <0.0001.

## Results

### Effect of air-drying on reduction of MNV-1, E. coli and L. innocua on contaminated tableware

#### Results for MNV-1

**Figure 2:**
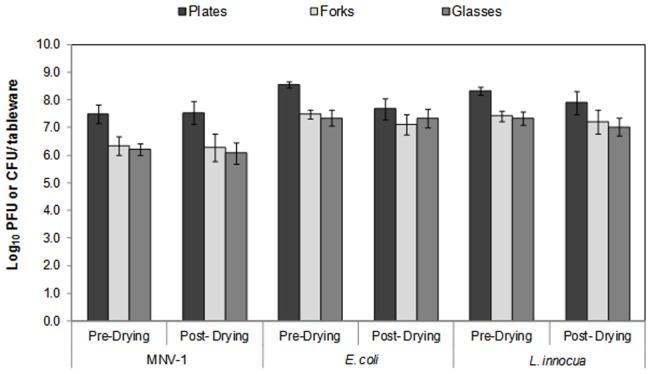
Survival of MNV-1, *E. coli* K-12 and *L. innocua* on different contaminated tableware before and after 1 hour air-drying at 24±2°C.

Fig. 2 shows the effect of this air-drying on the initial MNV-1titers. The highest mean reduction in the viral counts was 0.1 log PFU per tableware item. The statistical analysis confirmed that the viral counts on the contaminated tableware items before and after the air-drying period were not significantly different (*p*>0.0001). This result is consistent with those of other researchers who showed that norovirus can survive for up to 30 days on stainless steel (Takahashi et al., 2011) [14] and 7 days on fecal contaminated surfaces [15], [16]. This suggests that MNV-1 is quite resistant to air-drying and that it could remain infectious on food contact surfaces for an extended period of time. We also determined the recovery rate for the swab-rinse method used in our study. Briefly, the MNV-1 stock (10^8^ PFU/ml) was seeded on the surface of each table ware and was air dried for 1 h. The virus was recovered by swab-rinse method as described in Materials and Methods, and the titer was determined by plaque assay. The recovery rate was calculated from the titer recovered from each food contact surface were divided by initial viral titer. The results showed that the recovery rates from fork and drinking glass were approximately 99.5% and 99.3%, respectively.

#### Results for bacterial species

Fig. 2 also shows the effect of the air-drying on the initial counts of *E. coli* K-12 and *L. innocua* cells. The highest reductions observed for *E. coli* K-12 (0.9 log) and *L. innocua* (0.4 log) were on the plates. The mean reductions on the forks and drinking glasses for both organisms ranged between 0 to 0.4 log. No significant differences (*p*>0.0001) were found for bacterial counts before and after the air-drying period. Overall, these results are in agreement with previous studies [10], [13], where *E. coli* K-12 and *L. innocua* showed stability under drying conditions. These results also show that *E. coli* and *L. innocua* are more sensitive to desiccation stresses than MNV-1.

### Comparison between efficacies of mechanical and manual ware-washing protocols and effect of the sanitizers

#### Results for MNV-1

**Figure 3:**
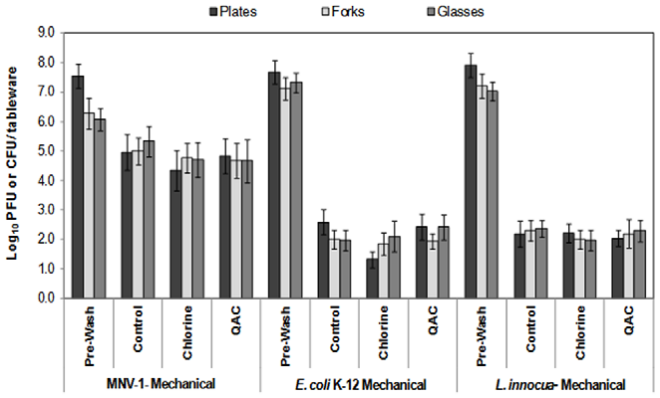
Survival of MNV-1, *E. coli* K-12 and *L. innocua* on contaminated tableware items after washing and sanitizing, using the mechanical dishwasher.

Fig. 3 shows the survivability of MNV-1 on the contaminated surfaces before and after mechanical washing. The results show that the mean reductions of MNV-1 on the plates, forks and drinking glasses after the washing treatment with the control were 2.6, 1.3 and 0.7 log, respectively. The mean reductions achieved after washing and chlorine sanitation (3.2, 1.5 and 1.4, respectively) were slightly higher than those obtained by the control treatment. Statistically, chlorine reductions were not significantly different (*p*>0.0001) from those achieved by the control. Similarly, the mean reductions achieved by the QAC sanitizer were not significantly different (*p*>0.0001) from the reductions produced by the control and chlorine treatments. The data showed that after washing and sanitizing with the QAC sanitizer, MNV-1 was reduced on the ceramic plates by 2.7 log and the mean reduction for both the forks and glasses were 1.6 and 1.4 log, respectively. Overall, the viral counts detected on the different surfaces after sanitization with the three treatments were statistically different (*p*<0.0001) than the initial viral counts prior to the ware-washing. When comparing the mean reductions achieved for MNV-1 on the three different surfaces, the data show that they were not statistically different (*p*>0.0001).

**Figure 4:**
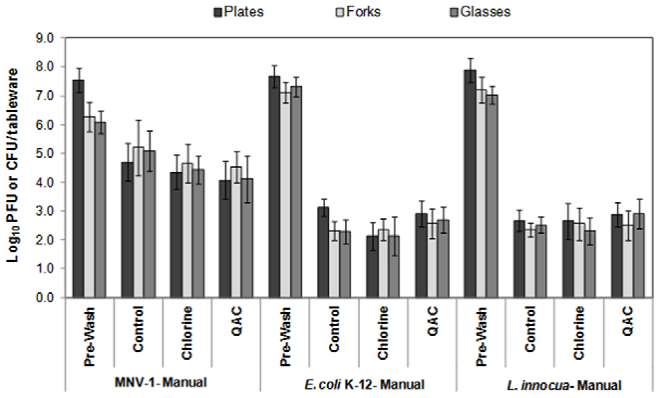
Survival of MNV-1, *E. coli* K-12 and *L. innocua* on contaminated tableware items after washing and sanitizing, during manual ware-washing.

The effect of the manual ware-washing and sanitizing solutions on the reduction of MNV-1 from the contaminated tableware items is presented in Fig. 4. For the control treatment, the mean reductions of MNV-1 on the plates, forks and glasses were 2.8, 1.1 and 1 log, respectively. The reductions achieved by the chlorine and the QAC sanitizers were slightly higher than the ones obtained by the control. For ware washing with chlorine sanitization, the reductions ranged from 1.7 to 3.5 log per tableware item. For the ware washing with the QAC sanitization, the range was 1.6 to 3.2 log per tableware item. The statistical analysis revealed that the mean reductions achieved after sanitization with the chlorine and QAC sanitizers were not significantly different (*p*>0.0001) than the reductions achieved by the control.

#### Results for bacterial species

The effect of the ware-washing and sanitizing solutions on the reduction of *E. coli* K-12 and *L. innocua* from the contaminated tableware items are also presented in Figs. 3 and 4. In general, the bacterial cells were significantly (*p*<0.0001) reduced after the tableware items were washed and sanitized. However, the data show that, the mechanical ware-washing produced slightly more inactivation of viable cells when compared with the manual method (Figs. 3 and 4, respectively). This could be attributed to the water pressure in the automatic dishwasher as well as the higher temperature used during the washing cycle. All sanitizing solutions during the mechanical ware-washing helped to produce ≥5 log reduction. Additionally, the results suggested that when the chlorine solution was used during mechanical ware-washing, the reduction of *E. coli* K-12 from the plates tended to be higher when compared with that of the forks and the drinking glasses.

## Discussion

Most documented foodborne viral outbreaks can be traced to food that has been manually handled by an infected food handler [17]. Hence, the hygiene of the personnel who handle food in foodservice establishments is an important preventive measure in minimizing cross-contamination of food contact surfaces and the food itself with norovirus [16], [18]. Additionally, the long persistence of norovirus on food preparation surfaces and its resistance to heat and disinfection, make the issue of cross-contamination reduction an even more urgent matter in the fight against foodborne outbreaks [16], [19].

Results presented in Figs. 3 and 4 reveal that even though sanitizers appear to slightly enhance the reduction of MNV-1 from contaminated tableware, there was still a considerable amount of the virus on the contaminated surfaces. Since norovirus is highly contagious and its infectious dose is relatively low (10–100 particles), only a few infectious virus particles can cause human infection [1], [3]. In accordance with the guidelines provided by the ANSI/NSF International standards (ANSI/NSF 3) and the FDA Food Code (2009) [11], any protocol used during ware-washing operation should achieve a 5-log microbial reduction. Unfortunately, these mandates are based on studies designed for the reduction of bacterial populations, but not viruses. Therefore, based on the results obtained in this present study, viruses such as MNV-1 seem to be quite resistant to the common sanitizers used in restaurants and other foodservice facilities. In addition to this, ware-washing protocols that previously showed effectiveness in removing a significant amount of bacteria from contaminated food contact surfaces [10] appear not to have the same efficacy for the removal of viruses.

**Figure 5:**
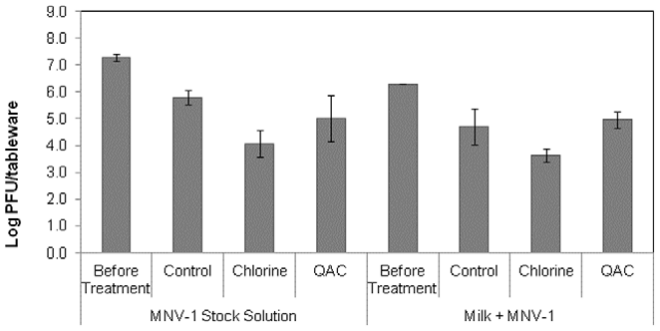
Inactivation of MNV-1 in stock solution and in inoculated milk by the Control, Chlorine (200 ppm) and QAC sanitizing (200 ppm) solutions at 49°C for 10 sec.

There are some possible explanations regarding the ineffectiveness of the ware-washing procedures to achieve higher reductions of the virus from the contaminated surfaces. One could be the food itself. Food residues are known to protect bacteria from direct contact with the heat or detergents used in dishwashing operations [20], [21], [13]. Likewise, inactivation studies suggest that food matrices may also provide a protective effect for virus inactivation [22], [23]. The food matrices used in our study were cream cheese and 2% reduced fat milk. Generally, milk and milk products are more difficult to remove from eating utensils than other types of foods [13] and they may act as protective agents. To support this, the protective effect of milk on MNV-1 against sanitizing solutions was also investigated (Fig. 5). In general, MNV-1 showed to be more sensitive to the chlorine sanitizer than to the QAC and control treatments when it was in suspension (virus stock) and in the milk. In fact, the chlorine sanitizer reduced MNV-1 (in stock solution) by 3.2 logs whereas the reductions obtained by the control and the QAC sanitizers were 1.5 and 2.3 logs, respectively. The MNV-1 mean reductions achieved with the chlorine sanitizer were statistically (*p*<0.0001) different than those achieved with the control treatment but not significant (*p*>0.0001) when compared with those of QAC. The efficacies of the sanitizing solutions were slightly reduced when the virus was present in the milk. The MNV-1 reductions achieved after exposure to the sanitizing solutions when the virus was in the milk were 1.6, 2.7 and 1.3 logs (control, chlorine and QAC, respectively). No significant differences (*p*>0.0001) were found between the control and the QAC sanitizer. However, the mean reductions achieved by the chlorine sanitizer were significantly different (*p*<0.0001) than those obtained with the control and the QAC. Another explanation for the persistence of MNV-1 on the surfaces could be the drying time prior to the experiment. All contaminated surfaces were allowed to dry for 1 h and it is possible that while slowly drying, they have formed a layer that protected the virus on the surfaces, resulting in a prolonged survival [19]. The formation of this layer can also be the reason for the detection of *E. coli* and *L. innocua* in our present study, even after ware-washing.

The ineffectiveness of the traditional sanitizers and the ware-washing protocols to significantly remove and/or inactivate MNV-1 from contaminated surfaces is in agreement with published reports which suggested that the current food hygiene guidelines, most of which have been optimized for the prevention of bacterial infections, may not be fully effective against viruses [17], [24]. Additionally, previous viral inactivation studies have also shown that non-enveloped viruses (e.g. noroviruses and their surrogates) are fairly resistant to chemical agents, including QACs and chlorine-based sanitizers [25], [26]. The poor virucidal activity of QAC against MNV-1 in our study could be attributed to its formulation and the types of microorganisms it is intended to kill. Whitehead and McCue, (2010) [27], and Nowak et al. (2011) [26], explained this by noting that when used alone, QAC sanitizers have limited effectiveness for inactivation of non-enveloped viruses such as norovirus and its surrogates. The literature also reports on studies using Feline Calicivirus (FCV) as a surrogate for human norovirus [15]. Although results from these studies differ from those obtained for MNV-1, in this present study, MNV-1 was chosen because it is more resistant to sanitization when compared with FCV. The use of MNV-1 in our studies thus presents a worst case scenario. This selectivity of QAC has been attributed to its ionic binding capabilities and hydrophobic interactions with microbial membrane surfaces. This is so because QAC is positively charged and when in contact with microorganisms its cationic head is oriented outwards and the hydrophobic tail attracted to the lipid bilayers of the organisms. This causes rearrangement of the membranes and subsequently leakage of the intracellular constituents [28]. However, the bactericidal activity of QAC can be reduced when bacteria, such as *E. coli* O26 and *Pseudomonas Aeruginosa* are allowed to dry with foods on surfaces. This is supported by a previous study conducted by Kuda et al., (2008) and Truby and Bennett (1996), where food sediments, such as milk, meat gravies, fats and certain carbohydrates are capable of adversely affected the bactericidal effect of QAC [29], [30]. These researchers also reported that this problem could be compounded if these organisms remain viable on the food contact surface and then go on to form biofilms which act to increase the protection of the microbes against sanitization.

The effectiveness of chlorine as a sanitizer is also limited by the action of organic matter [31], [32], [33]. In this study, the organic matter would be the food soil deposited onto the table ware items. Despite this, the results from our study showed that during both mechanical and manual ware-washing protocols, chlorine showed little effectiveness in reducing the MNV-1 on the contaminated surfaces. However, chlorine was able to inactivate both *E. coli* K-12 and *L. innocua*. The 200 ppm chlorine concentration that was used in the study represents the maximum concentration allowed by the FDA for sanitization of food contact surfaces [34]. Exceeding this concentration might have increased the reduction of both test virus and bacteria on the surfaces but could subject consumers to residual toxicity if used in a real world environment.

## Conclusions

From the results of our study, it could be concluded that QAC and sodium hypochlorite sanitizers normally used to inactivate bacteria in manual and mechanical ware-washing operations were unable to produce the same level of virus inactivation under similar conditions, irrespective of the nature of the tableware item tested. Further studies are needed to develop more effective ware-washing protocols for the removal of viruses from food contact surfaces/tableware items. Also, the combination of different detergents and sanitizing solutions (especially those containing surfactant agents) should be evaluated since they may help to enhance the removal and inactivation of non-enveloped viruses.
